# Change in the geometry of positive- and negative-powered soft contact lenses during wear

**DOI:** 10.1371/journal.pone.0242095

**Published:** 2020-11-09

**Authors:** Bartlomiej J. Kaluzny, Joanna Stachura, Patryk Mlyniuk, Alfonso Jimenez-Villar, Magdalena Wietlicka-Piszcz, Ireneusz Grulkowski

**Affiliations:** 1 Division of Ophthalmology and Optometry, Department of Ophthalmology, Collegium Medicum, Nicolaus Copernicus University, Bydgoszcz, Poland; 2 Institute of Physics, Faculty of Physics, Astronomy and Informatics, Nicolaus Copernicus University, Toruń, Poland; 3 Department of Theoretical Foundations of Biomedical Sciences and Medical Information Technology, Collegium Medicum, Nicolaus Copernicus University, Bydgoszcz, Poland; Keio University School of Medicine, JAPAN

## Abstract

Contact lens wear causes mutual interactions between the ocular surface and the lens, which may affect comfort as well as vision. The aim of this study was to examine deformations in modern positive- and negative-powered silicone hydrogel soft contact lenses (SiH SCLs) after 7 days of continuous wear. This pre-post interventional study included 64 eyes: 42 eyes with myopia of -3.00 D and 22 eyes with hyperopia of +3.00 D. All patients underwent general ophthalmic examination, corneal topography/tomography, total corneal and epithelial thickness mapping, and specular microscopy before and after the wearing period. SiH SCLs made of senofilcon A were worn continuously for 7 days on all eligible eyes. The geometry of the new and used lenses was measured 3 to 6 minutes after removal in two perpendicular planes using a custom-made swept source optical coherence tomography (SS-OCT) system for *in vitro* measurements. The anterior and posterior radii of curvature decreased in -3.00 D lenses in two perpendicular planes. This effect correlated significantly with average keratometry of the cornea. Sagittal lens height was lower in +3.00 D lens after wear, which correlated moderately with the corneal sagittal height. A significant decrease in central corneal epithelial thickness was observed after wearing +3.0 D lenses. In conclusion, SiH SCLs made of senofilcon A undergo minor deformations after 7-day continuous wear. Geometry modifications are different for -3.00 D and +3.00 D lenses, and they imitate the shape of the anterior eye surface. These geometric changes are accompanied by a decrease in the central thickness of corneal epithelium after +3.00 D lens wear.

## Introduction

More than 140 million people worldwide are estimated to wear contact lenses [1]. Soft contact lenses (SCLs) make up approximately 90% of current fits and refits, and the rest include rigid or hybrid contact lenses [2]. Contact lens wear causes mutual interactions between the ocular surface and the lens, inducing mechanical, biological, and chemical mechanisms, including hypoxic stress, that are associated with discomfort and may lead to vision-threatening conditions [3, 4]. Therefore, interaction between the eye and the contact lens impacts clinical tolerance of the lens.

Wearing SCLs can affect the anterior corneal curvature [5] but little is known about the modification of SCL shape during wear on the ocular surface. The effects of changes to SCLs during wear have been addressed in a few reports and include characteristics such as lens surface roughness [6, 7], the refractive index [8, 9] and lens shape [10, 11]. The authors observed a reduction in the overall lens diameter and the radius of curvature of the back optical zone in conventional hydrogel SCLs. The observed effects were linked to the dehydration of lenses during wear. However, modern silicone hydrogel (SiH) SCLs generally have lower water content and are less prone to variations in hydration [12, 13]. In these studies, the methodology involved an instrument based on mechanical measurement of the projection of the lens immersed in physiological NaCl solution, which limited the entire evaluation to the central thickness of the lens, the total diameter of the lens, and the radius of curvature of the back optical zone [10, 11].

New technology based on optical coherence tomography (OCT) was recently introduced for geometric inspection of SCLs. OCT has been very successful in ophthalmic diagnostics and offers a number of potential advantages over existing methods of lens inspection, including the ability to measure both sagittal depth and the thickness profile of the lens, and the ability to provide high-resolution geometric information across the entire soft lens immersed in temperature-controlled measurement solution [14–16]. The first commercial instrument based on spectral domain OCT was validated recently [17]. This new modality allows more comprehensive analysis of geometric changes in SCLs during wear, an issue that has very rarely been examined.

The aim of this study was to determine wear-induced deformations of SiH SCLs using OCT. To the best of our knowledge, the hypothesis that the change in SiH SCL shape after wear may have plastic characteristic and may be influenced by the geometry of the ocular surface of the eye has not yet been verified. This information may provide a scientific confirmation of everyday observations of the practitioners and could be useful in developing new materials and designs for contact lenses, especially in the face of an incipient revolution in contact lens developments, including drug-eluting lenses or lenses containing electronic components (e.g., sensors) [18–22].

## Materials and methods

### Study design

This is a prospective, pre-post interventional study conducted at the Division of Ophthalmology and Optometry, Department of Ophthalmology, Collegium Medicum, Nicolaus Copernicus University in Bydgoszcz, Poland, in accordance with the principles of the Declaration of Helsinki, the International Conference on Harmonization Good Clinical Practice guidelines, and all applicable laws and regulations. The study was approved by the Ethic Committee on Clinical Investigation (Institutional Review Board) at Nicolaus Copernicus University, and informed written consent was obtained from all patients before performing the measurements.

### Participants

The study involved two groups of contact lens wearers. Forty-two eyes in 25 participants (17 women and 8 men) with myopia of -3.00 D were enrolled in the myopic group, and 22 eyes in 13 patients (6 women and 7 men) with hyperopia of +3.00 D were enrolled in the hyperopic group (Table 1). The eyes with corneal or refractive astigmatism >1.5 D were excluded from the study. All patients were experienced contact lens users, without any ocular diseases or serious contact lens-related complications in their history. Minimal duration of SCL cessation before the initial examination was 7 days.

**Table 1:** Baseline characteristics of the enrolled eyes.

Characteristic	Myopic group (-3.00 D)	Hyperopic group (+3.00 D)	p
	n = 42	n = 22	
Age, years	23.5 (22–25)	36.5 (21.5–45.5)	0.071
Keratometry average, mm	7.69 (0.17)	8.00 (0.28)	<0.001
Horizontal visible iris diameter, mm	12.19 (11.92–12.51))	12.08 (11.96–12.37)	0.369
Corneal sagittal height, mm	3.43 (0.27)	3.12 (0.24)	<0.001
Sagittal height of the anterior segment, mm	4.51 (0.32)	3.97 (0.23)	<0.001
Central corneal thickness, μm	548.1 (3.4)	544.7 (23.7)	0.843
Central epithelial thickness, μm	51.9 (3.6)	53.3 (3.8)	0.199
Endothelial cell count, /mm^2^	2855 (256)	2663 (305)	0.021

Data are given as mean (standard deviation) or median (Q1 –Q3)

### Study protocol

All patients underwent a general ophthalmic examination, corneal topography/tomography with a Placido / Scheimpflug instrument (Sirius, CSO, Italy), total corneal thickness and epithelial thickness mapping with OCT (Avanti RTVue XR, Optovue, USA), and specular microscopy (EM3000, Tomey, Germany). The Placido / Scheimpflug instrument provided the horizontal visible iris diameter (HVID), corneal sagittal height (CSH), and anterior segment sagittal height (ASSH). CSH and ASSH were measured manually at HVID and a chord of 16 mm, respectively Subsequently, all eligible eyes wore SiH SCLs (Acuvue Oasys, Johnson and Johnson, USA) continuously for 7 days (-3.00 D in myopic group or +3.00 D in the hyperopic group; base curvature [BC] 8.4 mm, diameter [DIA] 14.0 mm). The lenses were made of senofilcon A, and the water content was 38%. The Young modulus was 0.7 MPa, central thickness for the -3.00 D lens 0.07 mm, and oxygen transmissibility Dk/t of the lens 147×10^9^. After the subjects stopped wearing the contact lenses, the eyes were examined again. Contact lens geometry was measured 3 to 6 minutes after removal in two perpendicular planes in a wet cell using a swept source OCT (SS-OCT) prototype [16]. The measurements of five new (freshly unpacked) contact lenses with the same design and power were considered a reference for statistical comparison.

### SS-OCT system and SCL measurements

**Fig 1:**
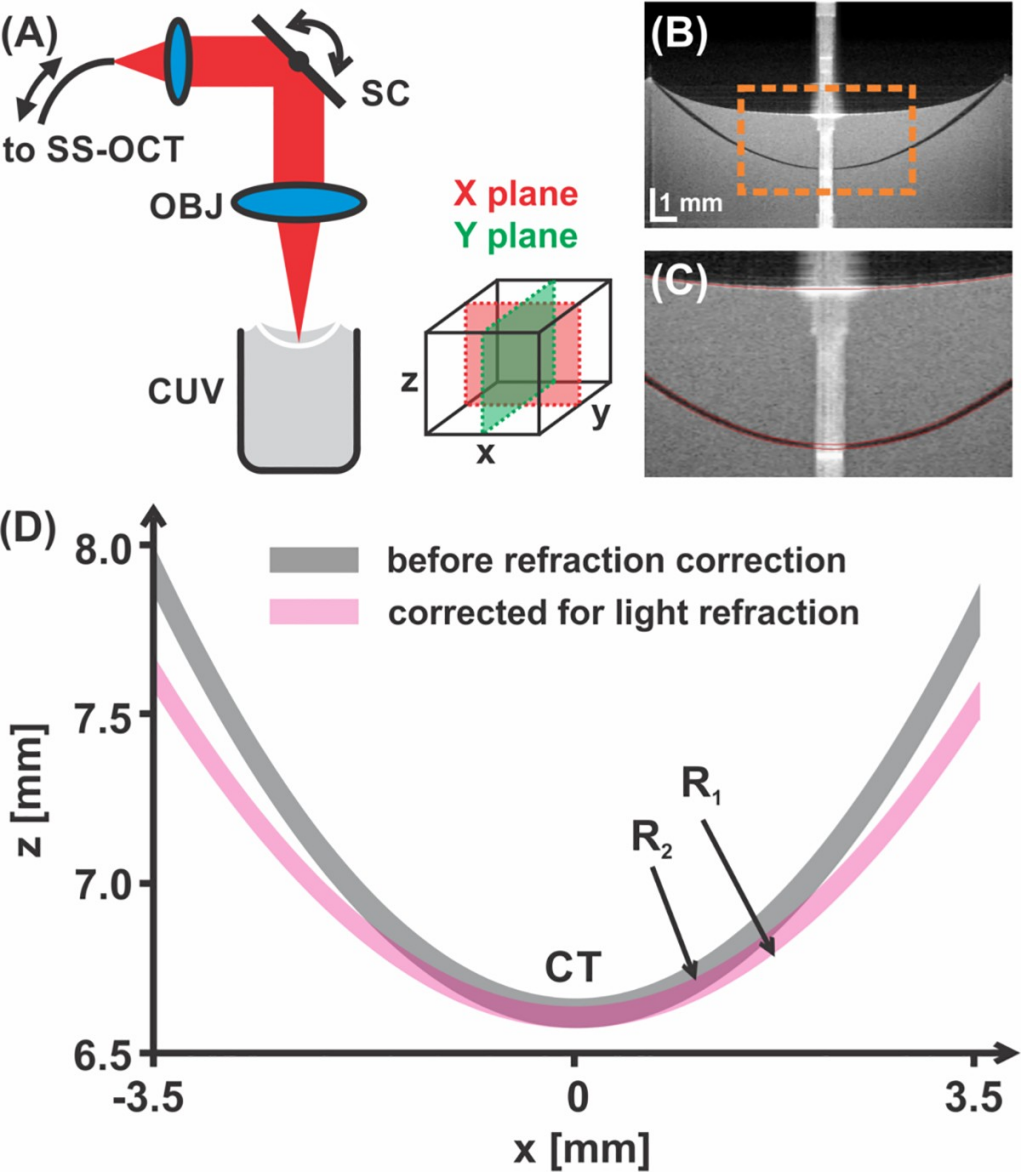
Experimental SS-OCT set-up for quantitative inspection of SCLs. SC, scanner; OBJ, objective; CUV, cuvette with the lens under investigation. (A) Central X and Y cross-section (plane) extracted from the three-dimensional data set. (B) Central cross-sectional image of the lens in the cuvette with enhanced contrast. (C) Segmentation of the intralipid solution interface and CL interfaces. (D) A refraction correction algorithm must be applied to the segmented surfaces to obtain geometrically accurate lens parameters. R_1_, anterior radius of curvature; R_2_, posterior radius of curvature; CT, central lens thickness.

The measurement procedure has been described in detail elsewhere [16]. The contact lenses were scanned using a custom-made SS-OCT system (Fig 1A) operating at a speed of 50 kHz and central wavelength of 1310 nm (Axsun Technologies Inc., Billerica, USA). Interference signals were digitized by a dual-channel acquisition board (Gage Applied Inc., Lockport, IL; Gage Compuscope 14200, 200 MS/s, 14-bit resolution), enabling a free-space depth (axial) range of 9.5 mm. A sensitivity of 110 dB was obtained when the sample was illuminated with a light beam with a power of 7.5 mW. The axial and transverse resolutions were 6 μm (in air) and 24.8 μm, respectively. The scan protocol allowed for generation of a three-dimensional (3-D) volumetric data set and was composed of 300 B-scans each of 300 A-scans (Fig 1A). The lens was placed in a wet cell filled with a 0.5% fat emulsion, made with Intralipid 10% (Fresenius Kabi, Germany) and 0.9% unpreserved saline solution at 20°C ± 0.5°C, which is in line with ISO 18369–3:2017 except the additional fat emulsion. An auto-positioning method based on the lateral capillary interactions between floating objects was utilized [16].

To obtain profilometric information about the SCL, the volumetric 3-D OCT data were post-processed during a multistage procedure (Fig 1B). First, central averaged sections (horizontal X and vertical Y scan) were extracted from 3-D data sets based on the *en-face* projection view (Fig 1A). The interface of the scattering solution and both contact lens interfaces were then segmented in both central cross-sections (Fig 1C). This allowed for a correction of the images for light refraction using a ray-tracing technique with the following refractive indices: intralipid solution, n_1_ = 1.378; senofilcon A, n_2_ = 1.42 measured before the study). The correction is crucial for obtaining the geometrically accurate shape of the imaged structures (Fig 1D). Finally, a best-fit spherical surface over a 6-mm-diameter central zone was used to measure the radii of the anterior and posterior curvatures in two perpendicular planes (X and Y) (Fig 1D). Before starting the actual study, a repeatability test of the method was performed by taking five measurements of new sample of Acuvue Oasys -3.00 D and +3.00 D SiH SCLs 3–6 minutes after removal from the blister. Repeatability was expressed by the coefficient of variation, defined by the relative standard deviation (standard deviation [SD] divided by the mean). All measurements were performed by a trained technician.

### Statistical analysis

Experimental data along with the results of statistical analysis are given in S1 Table. The summary statistics for normally distributed continuous variables are presented as mean and SD or as median and interquartile range for non-normally distributed variables. The normality of distribution was assessed by visual inspection of histograms and by the Shapiro-Wilk normality test. The differences between continuous normally distributed variables were analyzed by the t-test for independent samples or dependent samples by repeated-measures ANOVA. In the case that data were not normally distributed, the differences were tested using the Wilcoxon test when the samples were independent or the Friedman test for dependent samples. Pearson’s correlation coefficient (r) was used to examine the dependencies between select continuous variables. The results were considered significant when p<0.05. Alternatively, the differences between normally distributed variables were also reported in terms of Bayes factor BF_10_ which quantifies the likelihood of the data under the alternative hypothesis relative to the likelihood of the data under the null hypothesis. Bayes factor is a measure of the evidence for the alternative hypothesis, compared to the null hypothesis [23, 24]. The statistical analysis was performed using R software version 3.0.3 (The R Foundation) [25].

## Results

**Fig 2:**
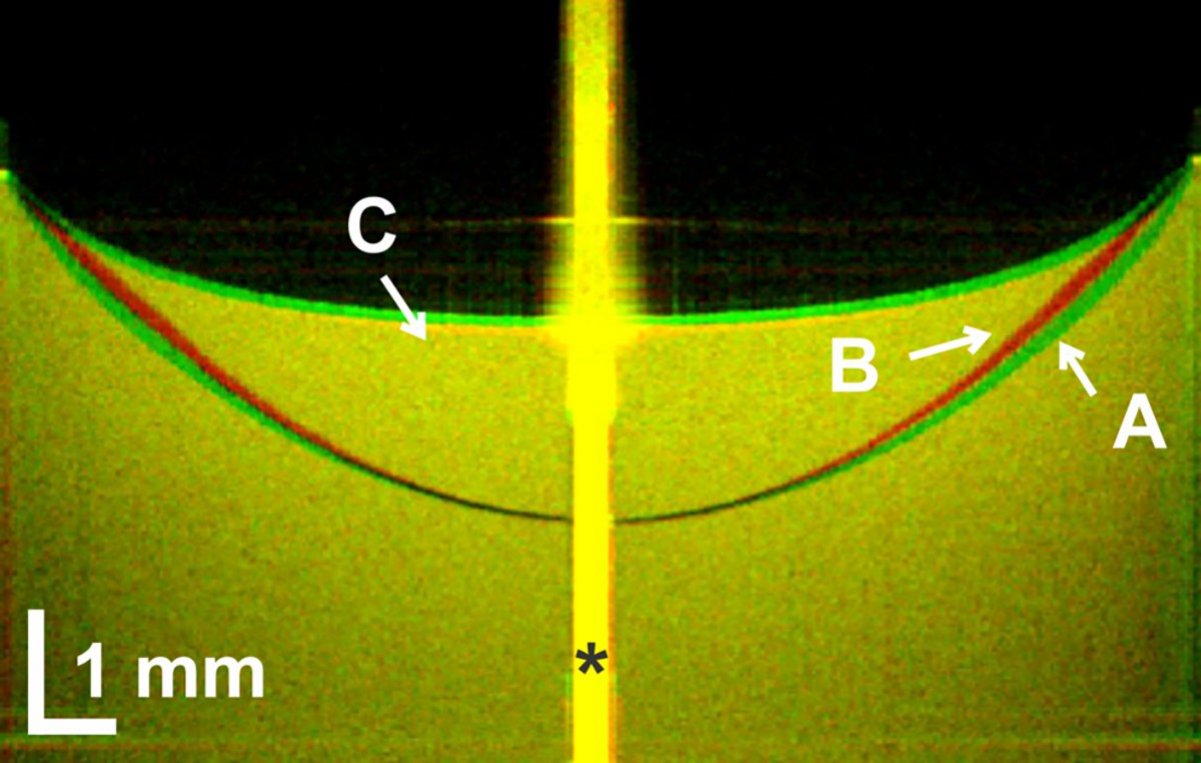
Superimposed cross-sectional SS-OCT images of a -3.00 D soft SiH SCL in a wet cell filled with solution of increased reflectivity. A—new lens, immediately after removal from the blister pack (green); B—used lens, immediately after removal from the eye after 7-day continuous wear (red); C—Meniscus of the solution of increased reflectivity. *artefact of the central light reflex.

Statistically significant differences in corneal keratometry, corneal sagittal height, sagittal height of the anterior segment and endothelial cell count (Table 1, S1 Table). Fig 2 presents two representative original OCT images of a new and used -3.00 D SCL immersed in the 0.5% fat emulsion in 0.9% saline solution. Overlaying both images emphasizes the differences in the lens geometries.

The measurements of new Acuvue Oasys -3.00 D and +3.00 D lenses are summarized in Tables 2 and 3, respectively, as “before”. The coefficients of variation for the new -3.00 D lens in two perpendicular planes were 0.16% and 0.08% for diameter, 1.5% and 0.77% for central thickness, 0.07% and 0.15% for sagittal height, 0.5% and 0.48% for anterior radius of curvature, and 0.86% and 0.56% for posterior radius of curvature (S1 Fig).

**Table 2:** Changes in the measurements of -3.00 D contact lenses in two perpendicular planes.

Parameter	Plane	Before (n = 5)	After (n = 42)	Difference	p
Diameter, mm	X	14.16 (14.16–14.16)	14.24 (14.19–14.31)		0.078
	Y	14.17 (14.17–14.17)	14.23 (14.17–14.32)		0.103
Central thickness, mm	X	0.055 (0.001)	0.052 (0.006)	0.003	0.001
	Y	0.056 (0.001)	0.051 (0.007)	0.005	<0.001
Sagittal height, mm	X	3.54 (3.54–3.54)	3.54 (3.52–3.56)		0.959
	Y	3.54 (3.54–3.55)	3.53 (3.5–3.55)		0.343
Anterior radius of curvature, mm	X	8.99 (0.05)	8.26 (0.26)	-0.73	<0.001
	Y	9.01 (0.04)	8.39 (0.24)	-0.62	<0.001
Posterior radius of curvature, mm	X	8.51 (0.07)	7.89 (0.25)	-0.62	<0.001
	Y	8.54 (0.05)	8.02 (0.22)	-0.52	<0.001

Data are given as mean (SD) or median (Q1 –Q3).

**Table 3:** Changes in the measurements of +3.00 D contact lenses in two perpendicular planes.

Parameter	Plane	Before (n = 22)	After (n = 22)	Difference	p
Diameter, mm	X	14.23 (14.23–14.25)	14.24 (14.12–14.33)		0.626
	Y	14.27 (14.27–14.32)	14.23 (14.14–14.31)		0.079
Central thickness, mm	X	0.099 (0.003)	0.101 (0.005)		0.648
	Y	0.1 (0.002)	0.101 (0.005)		0.576
Sagittal height, mm	X	3.52 (3.51–3.52)	3.43 (3.41–3.44)	-0.09	<0.001
	Y	3.52 (3.51–3.52)	3.42 (3.41–3.44)	-0.10	<0.001
Anterior radius of curvature, mm	X	8.16 (0.30)	8.04 (0.26)		0.165
	Y	8.16 (0.28)	8.21 (0.26)		0.632
Posterior curvature, mm	X	8.51 (0.21)	8.41 (0.26)		0.207
	Y	8.50 (0.16)	8.53 (0.28)		0.694

Data are given as mean (SD) or median (Q1 –Q3).

After 7 days of continuous wear, we found a significant decrease in the anterior and posterior radii of curvature of -3.00 D SCLs (8.99 mm vs 8.26 mm, with BF_10_ > 150, which indicated a very strong evidence for alternative hypothesis). However, the diameter and sagittal height did not change significantly (Table 2, S1 Fig). A slight increase in the central lens thickness was also observed. We also performed correlation analyses to study the changes in the measured parameters of the -3.00 D lens in two perpendicular planes and the corneal geometry. We found a significant correlation between the changes in the anterior and posterior curvature of the lens measured in the X and Y planes with the average keratometry (K_ave_). For the anterior curvature in planes X and Y, the correlation coefficient was r = 0.43 (p = 0.005) and r = 0.44 (p = 0.003), respectively, and in the posterior curvature it was r = 0.5 (p = 0.001) and r = 0.5 (p = 0.001), respectively (S1 Fig).

In the hyperopic group, we found a significant decrease in the sagittal height of the lens with BF_10_ > 150 indicating a very strong evidence for alternative hypothesis), but no change in diameter, central thickness, or central curvature after 7 days of continuous lens wear (Table 3, S2 Fig). Correlation analyses revealed a significant correlation between corneal sagittal height and the change in lens sagittal height measured in the X and Y planes (r = 0.44, p = 0.039 and r = 0.51, p = 0.015, respectively). In addition, we found a significant correlation between the change in lens diameter and K_ave_. For planes X and Y, r = 0.49 (p = 0.002) and r = 0.57 (p = 0.006), respectively, though the change in the median lens diameter was not significant (S2 Fig).

Table 4 presents the modifications in basic corneal parameters after 7 days of continuous wear. A significant decrease in the central epithelial thickness of 2.36 μm was observed after wearing the +3.00 D lens. The Bayes factor BF_10_ = 45, which indicated a strong evidence for alternative hypothesis. We also measured a slight but significant decrease in the endothelial cell count in the myopic group (S1 Table, S3 Fig).

**Table 4:** Changes in basic corneal parameters after 7 days of continuous wear.

Parameter	Myopic group -3.00 D				Hyperopic group +3.00 D			
	Before wear	After wear	Difference	p	Before wear	After wear	Difference	p
	Mean (SD) n = 42	Mean (SD) n = 42			Mean (SD) n = 22	Mean (SD) n = 22		
Keratometry average, mm	7.69 (0.18)	7.73 (0.36)		0.431	8.00 (0.28)	8.00 (0.27)		0.919
Central corneal thickness, μm	548.1 (30.4)	545.7 (29.6)	-2.4	0.042	544.7 (23.7)	543.2 (24.0)		0.206
Central epithelial thickness, μm	51.88 (3.59)	52.48 (4.10)		0.243	53.27 (3.75)	50.91 (3.33)	-2.36	0.001
Endothelial cell count, /mm^2^	2855.5 (256.1)	2799.3 (260.4)	-56.1	0.017	2662.9 (304.8)	2679.1 (308.4)		0.329

## Discussion

Two or three decades ago, the fitting of SCLs made of hydrogel was much more complicated than the current procedure. A practitioner had to select an appropriate diameter and base curve to fit the eye. The majority of SCLs are currently manufactured in only one diameter and base curve, and they are supposed to fit more than 95% of eyes. Consequently, we investigated whether modern SCLs adjust their shape to the shape of the ocular surface on which the lens is worn. One can also speculate that this adjustment is related to the contact lens design, mostly the thickness profile, as well as the mechanical properties of the material used for manufacturing the lenses.

The lenses investigated in this study are characterized by the same geometrical descriptors like base curve and diameter. SCLs were of opposite power (±3.00 D), which means that positive-powered SCL is centrally thicker than negative-powered SCL. The mechanical properties of the contact lenses are described by tensile (Young’s) modulus of the lens material [26]. The tensile modulus of modern SCLs is within a range of 0.3 to 1.5 MPa. Although these values come from *in vitro* measurements at room temperature (20°C), they demonstrate a correlation with the clinical performance of the lens. Higher tensile modulus values are more likely to cause mechanically driven adverse events during SCL wear, such as mucin ball presence, corneal erosions, superior epithelial arcuate lesions, lid wiper epitheliopathy, SCL-induced papillary conjunctivitis, and meibomian gland dropout [27]. SCLs made of senofilcon A used in this study are characterized by moderate tensile modulus (0.7 MPa). Senofilcon A belongs to the second generation of SiH materials in which hydrophilic properties were introduced through an internal wetting agent, poly(vinyl pirrolidone) [12]. Accordingly, the properties of the SiH lens material enable better comfort and acceptance by the lens wearers.

In this study, we observed deformations of the SCLs as the result of continuous lens wear. It has to be pointed out that the stability (and mobility) of the lens resting on the eye depends on the complex balance between the forces at the interfaces between tear film, the lens and the cornea [28]. During everyday usage, a repeated force is applied to the SCL by the eyelids with each blink, which causes reaction of the lens in terms of its instantaneous deformation [29]. In addition to that, the eyelid exerts constant pressure on the cornea when the eye is closed. It has been estimated that the eyelid pressure on the cornea is age-dependent and ranges between 5 and 30 mmHg, and the normal loads are in the millinewton (mN) range [30–33]. Therefore, simulation of mechanical loads during wearing period may help in explaining the measured effects. Other physical factors contributing to the lens deformations include hydration, osmolarity and temperature.

After 7 days of continuous lens wear, a significant decrease was measured in the anterior and posterior radii of curvature of a -3.00 D contact lens within a central 6-mm-diameter zone. Moreover, we found a moderate correlation between the amount of the decrease in the central curvatures of the lens and K_ave_. The steeper the anterior surface of the central cornea on which the lens was worn, the higher the deformation of the contact lens. On the other hand, we did not observe any change in the central curvatures of the +3.00 D lens. This can be explained by the flatter anterior corneal curvatures in the hyperopic group (see Table 4), or more likely by the differences in the lens thickness in the center. Central thickness measured for -3.00 D lens was 66 μm and decreased after wear to 61 μm, whereas for +3.00 D lens it was 100 μm and the change was statistically insignificant. Both permanent eyelid pressure during the night and intermittent pressure with blinking may contribute to changes in the shape of thin parts of SCLs, whereas the geometry of thicker fragments remains unaffected. Thick lens power formula can be applied to estimate how the observed modifications of SCL parameters influence the refractive power of the contact lens. The calculations showed that the absolute value of the power change did not exceed 0.26 D. However, the impact of contact lens geometry changes on visual acuity could be fully determined if one takes into account additional factors such as lens material modifications and accumulation of deposits affecting the stability of pre-lens tear film.

We did not observe any significant change in the diameter and sagittal height of -3.00 D lenses after 7 days of continuous wear. It could be explained by flattening of peripheral zones of the lenses. For +3.00 D lenses, we noticed a decrease in the median sagittal height. Moreover, we found a significant correlation between corneal sagittal height and the change in the sagittal height of the lens. The lower the sagittal height of the cornea, the higher the decrease in lens sagittal height. The direction of changes suggests that changes to the shape of +3.00 D lenses occur to adjust to the low sagittal height of the hyperopic eye.

The total diameter of the SiH SCLs made of senofilcon A did not change after wear. However, the dispersion of the measurements increased after the wearing period, and we found a significant correlation between changes in the diameter of the lens and K_ave_ in the hyperopic group. The flatter the cornea, the greater the increase in total lens diameter. Inversely, a decrease in lens diameter was observed on steeper corneas. Previous studies on short-term wearing of -3.00 D high water-content hydrogel SCLs reported a reduction in the overall diameter of the lenses and a reduction in the radius of curvature of the back optical zone radius [2, 10, 11]. The change in diameter was evaluated *in vivo* using a video slit lamp. The authors linked these changes with lens dehydration during wear. The water content of senofilcon A lenses was 38% and changes in SiH SCL shape may also be caused, to some extent, by the change in hydration of the lens material, however stable diameter does not support this hypothesis. Ozkan at al. reported a decrease of both diameter and base curve equivalent of variety of SiH SCLs immediately upon removal measured at 35˚C. The reference measurements were made at 21˚C and the authors conclude that the difference was mainly the result of the increase of the temperature from 21˚C to 35˚C [34].

The study was performed for the SCLs with refractive powers of +/-3.00 D. The study was designed to recruit groups of hyperopic and myopic eyes with symmetric absolute refractive errors. Although it is difficult to extend the results to the lenses with different range of power, one can speculate that the modifications of thinner and thicker SCLs might be larger. The hyperopic lenses with positive diopter have the thickest section at the optical axis and should be the least affected by the external force. The observed SCL’s impact on the cornea can be also especially important for the contact lenses with higher positive power values, which cause thinning of the central corneal epithelium.

The lenses were measured after 3–6 minutes of rest in 0.9% saline solution to achieve proper measurement temperature and hydration. The measurements of new and used lenses were performed in line with ISO standards, except the addition of fat emulsion to increase reflectivity during OCT measurements, which may be considered a limitation of the study. We expected that keeping the lens in this solution for a prolonged period of time may cause some changes in the lens material, which may also lead to shape changes. Therefore, we did not repeat the measurements after several hours or days.

Quantitative description of the shape changes of SCLs was performed on two perpendicular sections taken from 3-D data sets. However, volumetric data can provide with more detailed insight into post-wear SCL topography alterations. Therefore, the approach used in this study can be regarded as simplification of the problem but it was the easiest option to detect general modifications of the curvature of the lenses.

Another interesting finding was a significant decrease in the central thickness of the corneal epithelium immediately after removing the +3.00 D lens after 7 days of wear. In addition, this change did not influence keratometry readings, which indicates that the change must have been uniform over the central 3 mm zone. Hong et al. reported a decrease in epithelial thickness in subjects after long-term wear of SCLs [35]. However, Lu et al. recently reported an increase in the central epithelial thickness after overnight wear of -3.00 D SiH SCLs [36]. In our cohort, we also observed some increase in epithelial thickness in the group wearing -3.00 D lenses, but it did not reach significance. For +3.00 D lenses, with a relatively thick center, the decrease was significant and resembled the effect of orthokeratology [37]. However, in the case of +3.00 D SCLs, thinning of the epithelium was smaller and over a wider area, which did not influence keratometry readings.

## Conclusions

SS-OCT system for *in vitro* measurements allows comprehensive analysis of SCLs geometry and provides measurements with high repeatability. Modern SiH SCLs made of senofilcon A undergo minor plastic deformations after 7 days of continuous wear. Both the anterior and posterior central radii of curvature of a -3.00 D lens decrease after wear, which is more distinct on steeper corneas. The change in central curvature is not observed for +3.00 D lenses, but the sagittal height of the lens is reduced in correlation with small corneal sagittal height. The geometric changes, to some extent, imitate the shape of the eye surface, and are accompanied by a decrease in the central epithelial thickness in case of +3.00 D lens wear.

## Supporting information

S1 Table Experimental data and results of statistical analysis. (XLSX)

S1 Fig Changes in the measured parameters of -3.00 D contact lenses. (a) Lens diameter. (b) Lens central thickness. (c) Anterior radius of curvature. (d) Posterior radius of curvature. (e) Sagittal height.
(TIF)

S2 Fig Changes in the measured parameters of +3.00 D contact lenses. (a) Lens diameter. (b) Lens central thickness. (c) Anterior radius of curvature. (d) Posterior radius of curvature. (e) Sagittal height.
(TIF)

S3 Fig Changes in corneal parameters after 7 days of continuous wear of soft contact lenses. (a) Keratometry (radius of curvature). (b) Central corneal thickness. (c) Central epithelial thickness. (d) Endothelial cell count.
(TIF)

S1 File Data analysis code. (PDF)
