# Supplementary figures and images for: Change in the geometry of positive- and negative-powered soft contact lenses during wear
Source: PLoS One. 2020 Nov 9;15(11):e0242095. doi: 10.1371/journal.pone.0242095 (PMC7652269; doi:10.1371/journal.pone.0242095)

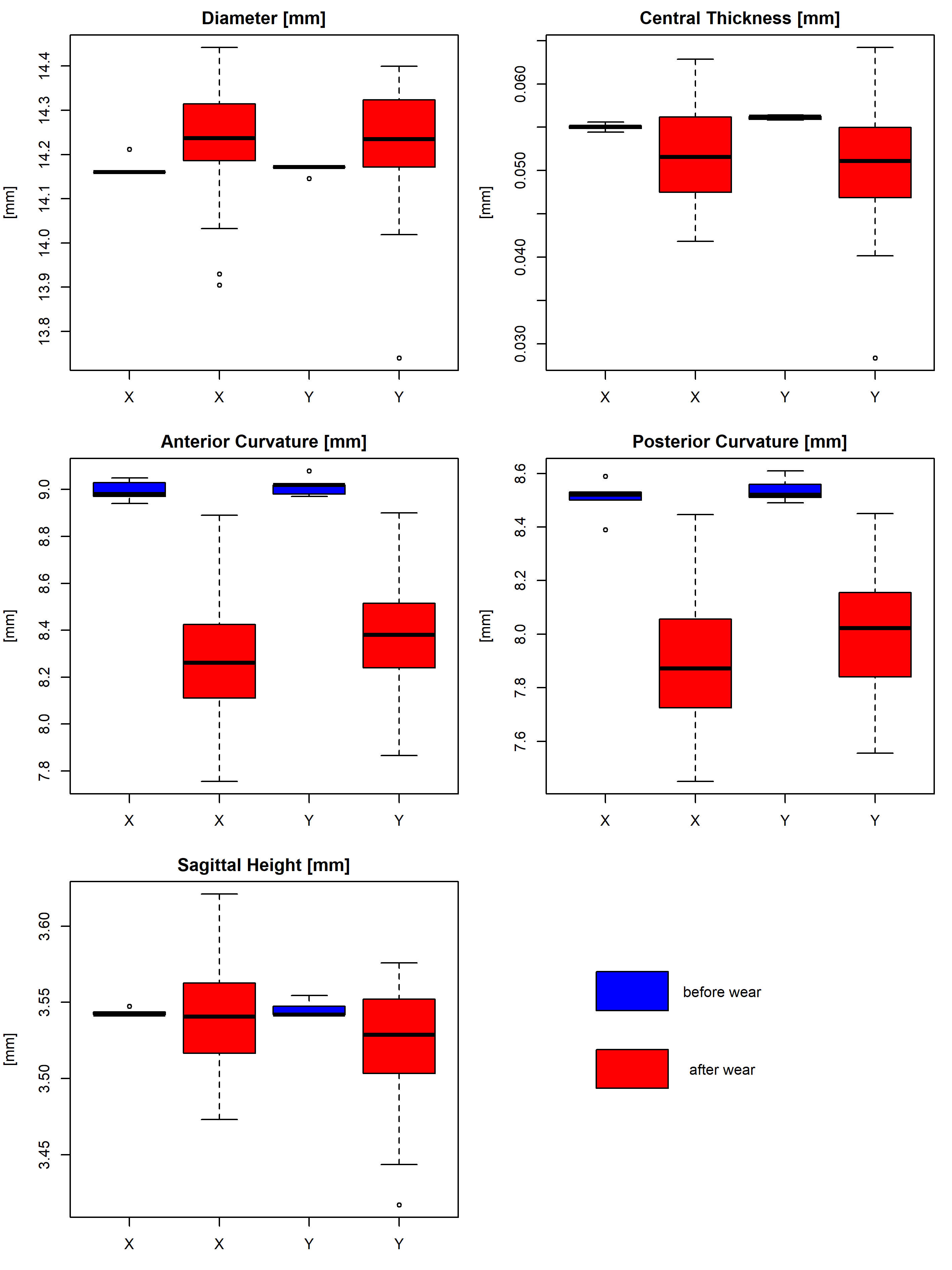

Supplement: S1 Fig — (a) Lens diameter. (b) Lens central thickness. (c) Anterior radius of curvature. (d) Posterior radius of curvature. (e) Sagittal height. (TIF) [file pone.0242095.s002.tif]

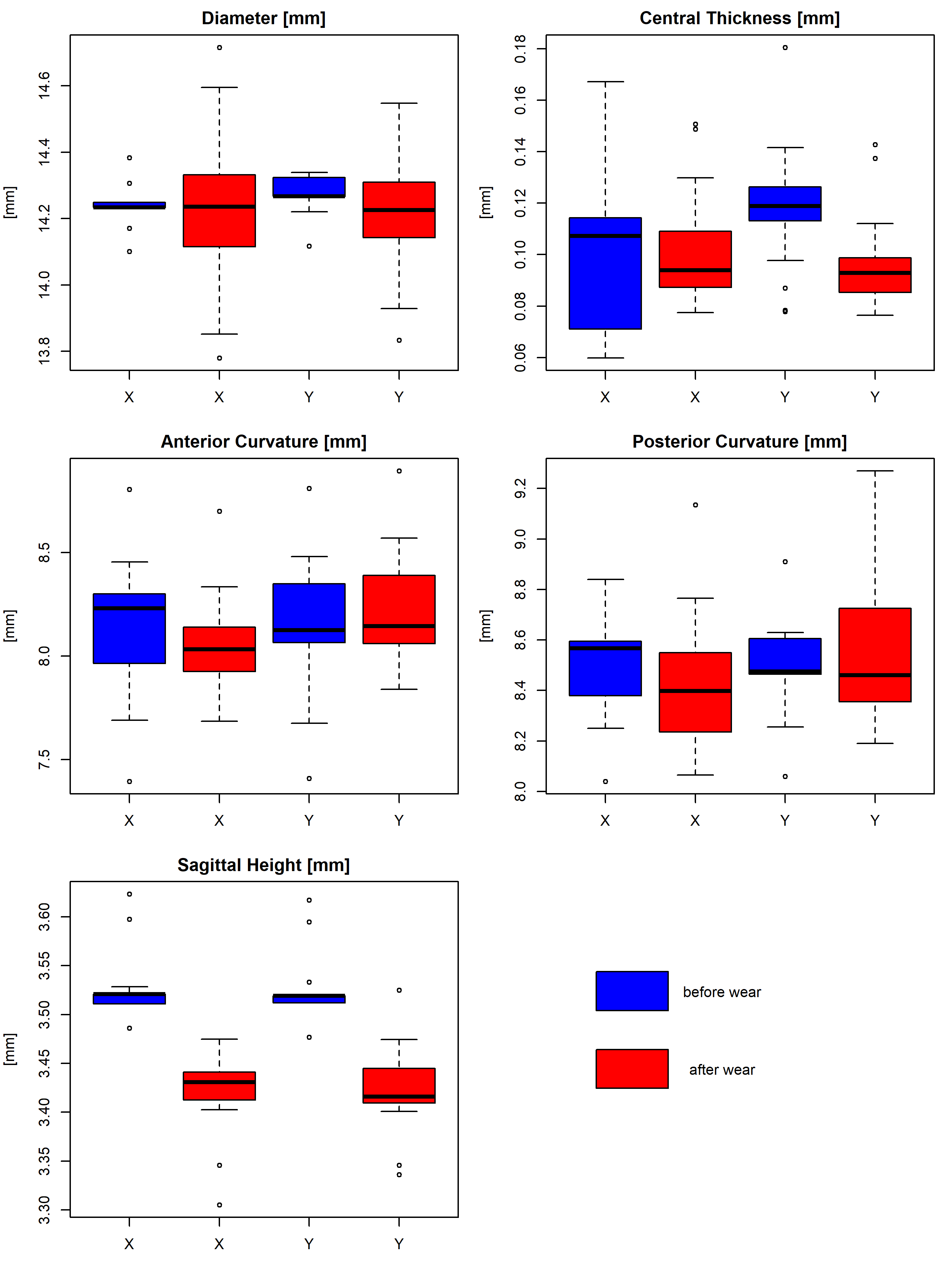

Supplement: S2 Fig — (a) Lens diameter. (b) Lens central thickness. (c) Anterior radius of curvature. (d) Posterior radius of curvature. (e) Sagittal height. (TIF) [file pone.0242095.s003.tif]

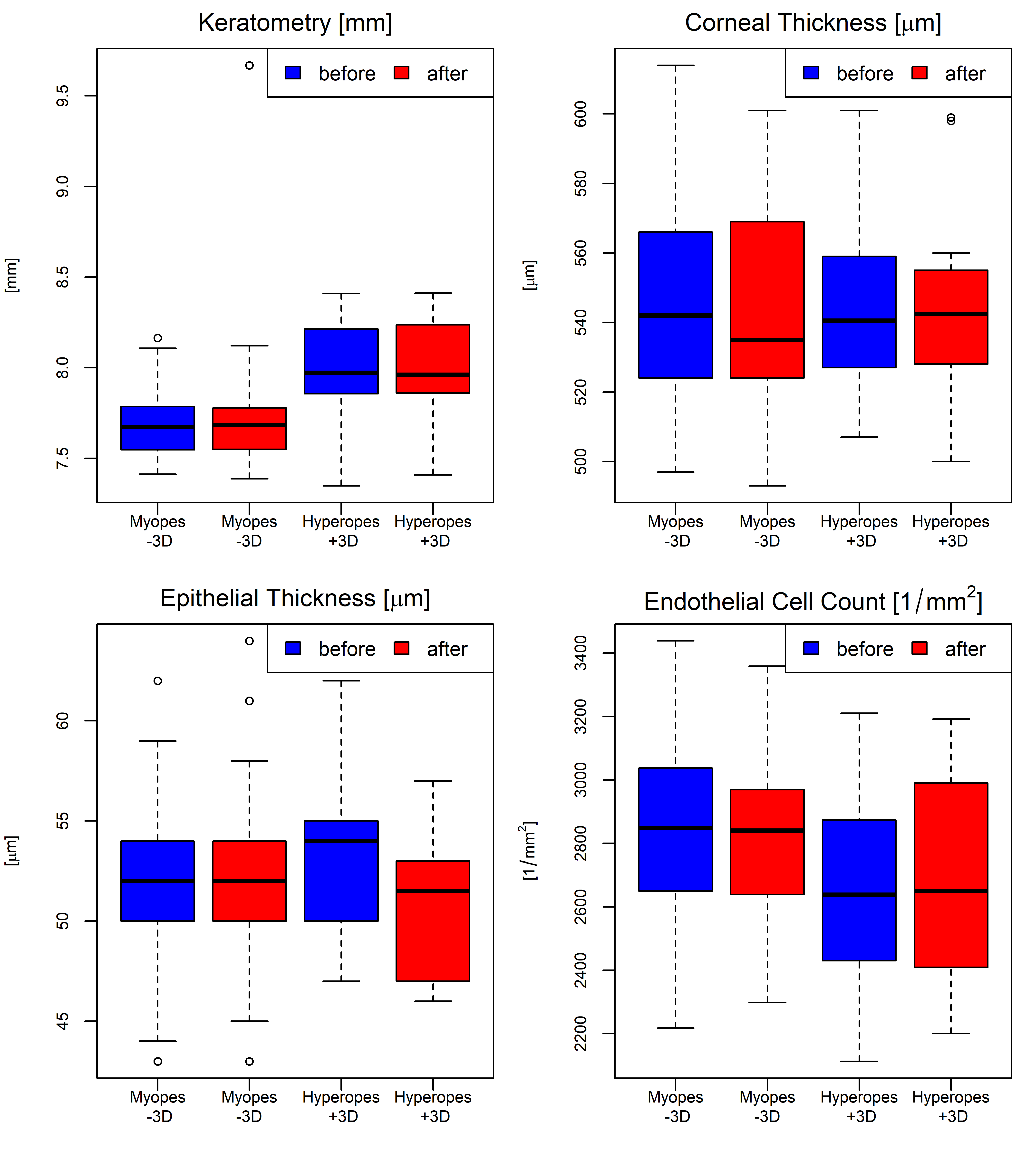

Supplement: S3 Fig — (a) Keratometry (radius of curvature). (b) Central corneal thickness. (c) Central epithelial thickness. (d) Endothelial cell count. (TIF) [file pone.0242095.s004.tif]
