# Supplementary material for: Change in the geometry of positive- and negative-powered soft contact lenses during wear
Source: PLoS One. 2020 Nov 9;15(11):e0242095. doi: 10.1371/journal.pone.0242095 (PMC7652269; doi:10.1371/journal.pone.0242095)
Supplement: S1 File — (PDF) [file pone.0242095.s005.pdf]

Code in R software

```
# testing for normality

# dane- the data.frame  containing data,   zm –investigated column of the data

shapiro.test(dane[,zm])$p.value;   shapiro.test(dane[,zm])$statistic

# descriptive statistics: mean, standard deviation, median, first and third quartiles

mean(dane[,zm])

sd(dane[,zm])

median(dane[,zm])

quantile(dane[,zm])[2]

quantile(dane[,zm])[4]


#testing for differences between groups:

# zm-the tested  column of the data,  zm_grup – the grouping variable

# t-test and Wilcoxon test

t.test(dane[,zm]~factor(dan1[,zm_grup]), paired=F) # for Table 4 paired=T

wilcox.test(dane[,zm]~factor(dan1[,zm_grup]))

# Bayes Factor

poziomy<- levels(factor(dane[,zm_grup] ))

ttestBF (x=dane[dane[,zm_grup]==poziomy[1],zm], y=dane[dane[,zm_grup]==poziomy[2],zm])
```
